# The Connections Between Dietary Fatty Acids, Inflammation, and Chronic Disease

**DOI:** 10.3390/nu17142322

**Published:** 2025-07-15

**Authors:** Megan L. Falsetta, Emanuelle Chrysilla

**Affiliations:** 1 Obstetrics and Gynecology, The University of Rochester, Rochester, NY 14642, USA; 2 Pharmacology and Physiology, The University of Rochester, Rochester, NY 14642, USA; emanuelle_chrysilla@urmc.rochester.edu

## 1. Introduction

This special issue contains 4 primary research articles and 2 reviews that together highlight the role of polyunsaturated fatty acids (PUFAs), particularly those derived from diet or natural products, in human health and disease. Panezai and van Dyke review the role of PUFAs in oral health [1], while Zhao et al. review the connections between gut microbiota metabolism of short-chain fatty acids and heart failure [2]. Höpfinger et al. provide primary evidence that oral lipid ingestion affects the levels of adipokines, specifically cathelicidin antimicrobial peptide (CAMP) [3]. Yao et al. demonstrate CCN1/integrin α_5_β_1_ drives hepatic lipotoxcity via NLRP3-dependent pyroptosis [4]. Crovella et al. show that enriching cell membranes with docosahexaenoic acid (DHA) can enhance the response to chemotherapeutic acid doxorubicin [5]. Oladele and Fischer et al. demonstrate that arachidonic acid (AA) helps to prime human vulvar fibroblasts for inflammatory responses, which occurs concomitantly with pro-resolving mediator deficits leading to chronic pain [6]. This editorial seeks to integrate the findings of these 6 articles and additional sources into a narrative discussing the effects of dietary PUFAs on human health.

## 2. Dietary PUFAs and Health

As early as birth, the fatty acids in breast milk begin to shape the human microflora and condition immune responses within the human body [2]. Dynamic interactions between the human microbiome, environment, diet, host factors, and foreign microbes help shape human health. When the balance shifts away from mutualistic or commensal relationships, inflammation ensues, which can have significant negative impacts on health. Chronic inflammation instigates or at least exacerbates an array of human diseases, such as heart disease, diabetes, metabolic syndrome (metS), fatty liver disease, and inflammatory bowel disease, often leading to chronic pain, reduced quality of life, and ultimately mortality [3,4].

Insufficient fiber intake and consumption of processed foods that are high in sugar, saturated fats, and salt, often referred to as high-calorie, low-impact foods, or the Western diet, can cause or exacerbate human disease, including diabetes, metS, and fatty liver disease [2,7,8,9]. Therefore, dietary modifications have been suggested as a potential intervention, often citing the benefits of the Mediterranean diet, which is high in plant based dietary fiber, limits consumption of red meat, and contains fats that are largely monounsaturated fatty acids derived primarily from olive oil [2]. In support, several studies have found improved health outcomes for those that adhere to the Mediterranean diet compared to those that consume the Western diet [10,11]. Other approaches have looked to dietary supplementation with polyunsaturated fatty acids with pro-resolving properties, such as eicosapentaenoic acid (EPA) and docosahexaenoic acid (DHA); several clinical trials have shown at least modest success in improving health outcomes, although the effects are often lost after discontinuing supplementation [12,13,14,15,16,17].

While omega-3 PUFAs (e.g., DHA, EPA) are considered pro-resolving and beneficial for health, omega-6 PUFAs, derived from arachidonic acid (AA), are associated with inflammation and disease [1]. The ratio of n-6 to n-3 fatty acids can affect inflammation and immunity with higher ratios of n-6/3 lowering immune cell function [1]. The Western diet n-6/3 ratio is currently 20-fold higher than it was 100 years ago [1], while metabolic and immune disorders are on the rise [18,19,20]. The potential benefits of omega-3 PUFAs appear to far-reaching; DHA enrichment in cell membranes can enhance the membrane signaling pathways that impart anticancer effects and can augment the effects of chemotherapeutic agents, such as doxorubicin [5]. However, only three preparations of PUFAs are currently approved for use by the FDA: Lovaza, Omytrg, and Vascepa [21].

## 3. PUFAs as Therapeutics

The evidence for oral supplementation with PUFAs is mixed for several reasons that we will discuss herein. However, the potential for positive effects may outweigh any negative effects, especially considering that many PUFA preparations are natural products and an existing part of most diets.

Oral dosing with fish, krill, and plant-based oils rich in EPA and DHA has been shown to reduce pro-inflammatory mediator levels in plasma and inflamed tissues in several randomized controlled trials [13,22,23,24,25]. Although some mediators may be more highly repressed, interleukin-6 (IL-6), tumor necrosis factor alpha, c-reactive protein, and leukotriene B4 are consistently inhibited by PUFA treatment across numerous trials [22,24,25], an effect that may be linked to reduced nuclear factor kappa B (NFκB) signaling, a transcriptional factor that plays a significant role in inflammatory responses [23]. When inflammation becomes chronic, it can cause or exacerbate disease, such that a reduction in inflammation should convey beneficial effects [26]. As an example, clinical trials of Lovaza, an oral capsule containing omega-3 acid ethyl esters derived from fish oil, demonstrated a reduction in serum triglycerides, leading to Lovaza’s approval by the FDA to treat patients with high triglycerides, or hyperlipidemia, a frequent precursor to cardiovascular disease [27].

However, oral administration of PUFA-enriched fish oil preparations has not been definitively demonstrated to prevent or alleviate cardiovascular disease events [28]. One potential limitation with oral supplementation with PUFAs is that they must go through first-pass metabolism before entering the bloodstream where they can eventually access inflamed areas [29]. The literature on PUFA availability in red blood cells, plasma, and tissue following oral dosing of experimental or FDA-approved preparations, such as Lovaza, shows uptake occurs, but it is variable; PUFA levels can also be impacted by other dietary and lifestyle factors [12,15,16,17,30,31,32]. However, oral dosing of PUFAs (e.g., EPA and DHA) universally increases the levels of PUFAs in red blood cells, plasma, and most organs and tissues [12,16,17,30,31,32]. Whether this is sufficient to impart the intended biological effects is less clear.

The STRENGTH trial hypothesized that a carboxylic acid formulation of omega-3 fatty acids (EPA and DHA; Epanova) could improve cardiovascular outcomes in statin-treated patients with high cardiovascular risk, high triglycerides, and low HDL cholesterol levels. However, the trial was stopped early due to a lack of an apparent effect [28]. Even with concomitant statin treatment, the patients selected for this trial had a high risk of cardiovascular events, which was the primary outcome measure. It is plausible that these patients were unlikely to benefit from PUFA supplementation, at least not in terms of fewer cardiac events, while patients with earlier stage disease and lower risk could have experienced a benefit. Selection of subjects and outcomes influences the apparent success of any clinical trial. Starting trials with a lower risk patient population and focusing on more attainable outcomes, such as increases in HDL and decreases in triglycerides, may have been more successful.

The source, quality, and stability of the PUFAs tested can also alter the effects of omega-3 rich oils. Concerns about the bioavailability of ester forms of PUFAs (e.g., Lovaza) led to the development of Epanova, a free fatty acid preparation [17,31]. Some reports suggested Epanova had superior bioavailability compared to Lovaza, while others demonstrated little or marginal improvement. Ultimately, phase III STRENGTH trials of Epanova were discontinued due to the low likelihood of benefit for patients with mixed dyslipidemia [28]. Other important considerations in natural product testing are batch effects, storage conditions, and long-term stability, which make standardization of natural products more challenging. In 2012, Vascepa, a synthetic EPA (icosapent ethyl), was approved for individuals with high triglycerides, which is currently used as an adjunctive therapy to statins [33]. More recent clinical trials demonstrated Vascepa’s efficacy in reducing ischemic events, including cardiovascular death, but only for at risk patients meeting specific criteria [34].

PUFAs have been researched in various contexts with mixed results. However, emerging evidence indicates that deficits in PUFA metabolism, which may be acquired or genetically inherited, can also reduce their efficacy. Several inflammatory illnesses, such as asthma, sepsis, diabetes, and chronic vulvar pain all appear to be at least exacerbated, if not caused by dysregulated PUFA metabolism and the ensuing lipid dysbiosis [35,36,37,38]. This is another factor that may have influenced the outcomes of clinical trials of PUFA supplementation. Lipids dysbiosis, beyond that which is clinically measured, may play a critical role in human disease.

From PUFAs, specialized pro-resolving mediators (SPMs) are derived, which have more potent pro-resolving effects [39]. However more than 20 years after their discovery, they have yet to be implemented clinically [40]. SPMs are unlike anti-inflammatory drugs, such as steroids and non-steroidal anti-inflammatory drugs (NSAIDs). They do not stop or inhibit inflammation, rather they hasten the resolution of inflammation. However, while effective at low (nanomolar) concentrations and highly safe, they are unstable and susceptible to damage from light, heat, and oxygen [41]. They are also not sufficiently abundant within dietary sources to be purified in quantities large enough to be applied commercially as natural compounds [42]. Rather, they require chemical synthesis, which transforms these safe, natural compounds into new drugs that require rigorous clinical testing. However, this also affords the opportunity to modify these natural compounds to enhance their stability and ultimately their shelf-life, which may help to foster their clinical translation [41].

## 4. Vulvodynia: A Little-Known Example of Lipid Dysbiosis

Localized provoked vulvodynia (LPV) is a type of chronic vulvar pain that lasts at least 3 months, often years or decades [26,43]. Pain is localized to the vulvar vestibule, or ring of tissue immediately surrounding the vaginal opening, which is provoked upon contact, often impairing the individual’s ability to wear tight pants, ride a bicycle, sit in a chair, use tampons or menstrual cups, or engage in penetrative sexual activity. While not in of itself life threatening, vulvodynia greatly diminishes quality of life and is associated with depression, relationship issues, and missed days from work and school. LPV was initially considered psychosomatic because of the lack of an identifiable cause or pathology [44]. At most, patients experience mild erythema, or reddening, of the tissue surrounding the vagina, which provides no diagnostic value [26,43,45,46]. Vulvodynia is only diagnosed by ruling out all other recognized causes of vulvar pain. However, more recent evidence has identified inflammation as a key player, if not the instigator, of vulvar pain [26,36,47,48,49,50,51,52]. Delineating these inflammatory mechanisms has already served to identify potential therapeutic targets and novel markers of disease [26,36,47,53]. Like many of the more well studied diseases mentioned thus far, lipid dysregulation appears to be the catalyst for LPV disease [26,36,53].

AA, derived from dietary linoleic acid, which is abundant in nuts and seeds, is converted into both pro-resolving and pro-inflammatory lipids via the actions of several key enzyme families [1,39]. AA is stored in cellular membranes and released by phospholipase A2 (PLA2). AA can then be metabolized by lipoxygenase (LOX), cyclooxygenase (COX), and cytochrome P45 (CYP450). Constitutively expressed COX-1 and inducible COX-2 convert AA into prostaglandins and thromboxanes, which are inflammatory. LOX consists of a family of four enzymes that are largely involved in producing pro-resolving mediators by converting AA to hydroxyeicosatetraenoic acids (HETEs) and then lipoxins, a class of SPMs. However, LOX activity can also result in the production of inflammatory leukotrienes, namely through the activity of 5-LOX. CYP450 is a family of nearly 60 enzymes largely involved in drug metabolism [54]. However, CYP450 activity also results in the production of epoxyeicosatrienoic acids (EETs), which are anti-inflammatory lipids that are essential in the resolution of inflammation.

In vulvodynia, leukotrienes and prostaglandins are elevated in tissue and cells from the painful vestibule compared to non-painful adjacent control tissue from each patient and to control tissue from patients without vulvar pain, while lipoxins, 12-HETE, 8(9)-EET and 14(15)-EET are significantly reduced [26,36,47,53]. This culminates in enhanced inflammation and reduced resolution capacity, leading to chronic inflammation. In painful vestibular tissue, mRNA expression of PTGS2 encoding COX-2 and ALOX5 encoding 5-LOX is elevated, while expression of ALOX15B encoding 15-LOX-2 is decreased [6]. There are no differences in the expression of ALOX12 and ALOX15, encoding 12-LOX and 15-LOX-1, respectively. However, total LOX enzyme activity is diminished in fibroblasts isolated from the painful vestibule, irrespective of gene expression in these cells, suggesting there could be a gene polymorphism that reduces 12-LOX activity resulting in insufficient production of 12 HETE and lipoxin.

The addition of surplus AA further exacerbates lipid dysfunction in vulvar fibroblasts, reflecting the inflammatory role of n-6 fatty acids [6]. While PUFA supplementation with DHA or Lipinova^®^, a fish oil extract enriched for SPM precursors, resolves pain in a mouse model of vulvodynia, it is unclear if this approach would be successful in patients [36,47]. Although DHA and Lipinova^®^ are generally regarded as safe and are available in over-the-counter dietary supplements, it is unclear if this would have a benefit for LPV patients for two reasons: (1) oral dosing may not result in local changes in the vulvar lipidome, and (2) PUFAs may not be sufficiently metabolized, even if provided in excess, to recoup the SPMs and pro-resolving lipids that are deficient in patients.

**Figure 1:**
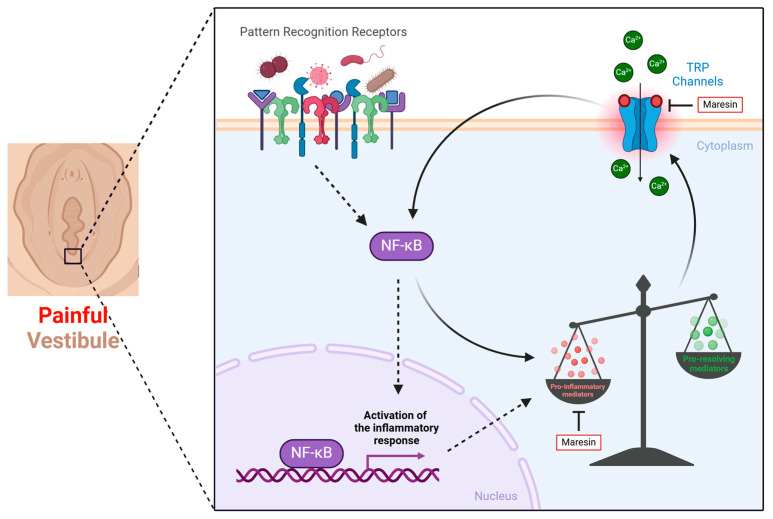
The “Perfect Storm” of Localized Provoked Vulvodynia.

Pain in LPV is at least in part caused by a series of linked events that involve microbes or other inflammatory insults, lipid dysregulation, and augmented pain signaling that converge into the “perfect storm” (Figure 1) [26,53]. First, the painful vestibule exhibits elevated expression of innate immune receptors known as pattern recognition receptors (PRRs) that recognize inflammatory stimuli, namely bacteria, fungi, and viruses [44,48,49,55,56]. The interaction of the resident flora appears to be enough to trigger an unnecessary inflammatory response, which alters the lipidome, resulting in the accumulation of pro-inflammatory mediators and lipids that initiate pain signaling through transient receptor potential (TRP) channels and potentially other yet unidentified pain channels. Pain signaling further augments inflammation, which further exacerbates lipid dysbiosis, and in turn amplifies pain signaling [26,36,53]. This cyclical process can be likened to a snowball rolling down a hill, enlarging with every rotation [26]. However, this cycle can be interrupted by supplementation with pro-resolving mediators, such as maresin 1, which not only reduce inflammation by compensating for the “missing” SPMs but also act directly on pro-nociceptive TRP channels to reduce pain signaling [36]. While this approach is highly successful in mice [36,47], we do not know if it will be successful in human trials. This approach would not rely upon conversion of PUFAs to SPMs, which would circumnavigate any deficits in PUFA metabolism. However, it will take years to complete the necessary safety and efficacy trials before it could be considered for approval by the FDA.

There has been a longstanding interest in the impact of diet on vulvar pain, specifically in reducing oxalates, which are excreted in urine and thought to potentially irritate vulvar skin [57,58,59,60,61]. However, there is no compelling evidence that supports this theory [57]. The handful of studies conducted to date show no significant benefit in testing for or trying to reduce urinary oxalate levels [58,59,60]. However, some patients have claimed to experience a reduction in their pain by reducing sources of dietary oxalates [61]. Interestingly, this diet also reduces the dietary intake of foods rich in linoleic acid, such as seeds and nuts. This is certainly not adequate evidence to support dietary changes in patients with LPV, but perhaps for some patients, reducing oxalates could reduce dietary sources of AA. It is worth considering when designing future studies to better understand the role of lipid dysbiosis in vulvodynia and the effects of dietary fatty acids.

In the painful vestibule, an exaggerated inflammatory response caused by increased pathogen recognition receptor expression leads to changes in the lipidome that activates TRP-associated pain signaling. This activation further elevates pro-inflammatory mediator production, which results in more TRP channel activation, thus creating a self-perpetuating cycle of inflammation and pain. Maresin 1 supplementation disrupts this cycle by reducing inflammation and directly inhibiting TRP channels, thereby promoting resolution. Figure created with BioRender.com.

## 5. Conclusions

While inflammation is a necessary response to protect the body from infection and injury, chronic inflammation is deleterious, contributing to numerous adverse health effects [26]. Polyunsaturated fatty acids and the SPMs derived from these PUFAs have pro-resolving effects that can effectively quell inflammation [13,22,23,24,25]. However, oral PUFA supplementation does not always translate to improved health outcomes, and the FDA-approved supplements have a limited scope of clinical application; they are largely approved to treat hyperlipidemia, but not the downstream cardiovascular effects of hyperlipidemia [17,21,22,28,29,30,33,34]. One explanation for the limited efficacy of PUFAs is the need to metabolize them into more highly bioactive lipids, a process that can be dysregulated in several diseases, such as vulvodynia [26,35,36,37,38]. Other considerations include formulation, application, primary and secondary trial outcome and subject selection, and bioavailability of the PUFAs or their metabolites at the site of inflammation. Using SPMs as a treatment is not a novel concept, but they have yet to be approved for clinical use due to several limiting factors, such as their instability, our inability to purify them from natural sources in sufficient quantities, and the need for rigorous clinical trials [39,40,41,42]. However, the existing evidence supports further investigation into their applications and the applications of other PUFAs or PUFA metabolites in treating diseases that are associated with chronic inflammation.
